# Decreased Chronic Morbidity but Elevated HIV Associated Cytokine Levels in HIV-Infected Older Adults Receiving HIV Treatment: Benefit of Enhanced Access to Care?

**DOI:** 10.1371/journal.pone.0077379

**Published:** 2013-10-15

**Authors:** Portia C. Mutevedzi, Alison J. Rodger, Paul Kowal, Makandwe Nyirenda, Marie-Louise Newell

**Affiliations:** 1 Africa Centre for Health and Population Studies, University of KwaZulu-Natal, Somkhele, South Africa; 2 University College London, Department of Infection and Population Health, London, United Kingdom; 3 World Health Organization Solid Action on Globalization and Environment, Geneva, Switzerland; 4 University of Newcastle Research Centre on Gender, Health and Ageing, Newcastle, Australia; 5 University of Southampton, School of Social Sciences, Highfield, Southampton, United Kingdom; 6 University College London Institute of Child Health, London, United Kingdom; University of Texas Medical Branch, United States of America

## Abstract

**Background:**

The association of HIV with chronic morbidity and inflammatory markers (cytokines) in older adults (50+years) is potentially relevant for clinical care, but data from African populations is scarce.

**Objective:**

To examine levels of chronic morbidity by HIV and ART status in older adults (50+years) and subsequent associations with selected pro-inflammatory cytokines and body mass index.

**Methods:**

Ordinary, ordered and generalized ordered logistic regression techniques were employed to compare chronic morbidity (heart disease (angina), arthritis, stroke, hypertension, asthma and diabetes) and cytokines (Interleukins-1 and -6, C-Reactive Protein and Tumor Necrosis Factor-alpha) by HIV and ART status on a cross-sectional random sample of 422 older adults nested within a defined rural South African population based demographic surveillance.

**Results:**

Using a composite measure of all morbidities, controlling for age, gender, BMI, smoking and wealth quintile, HIV-infected individuals on ART had 51% decreased odds (95% CI:0.26-0.92) of current morbidity compared to HIV-uninfected. In adjusted regression, compared to HIV-uninfected, the proportional odds (aPOR) of having elevated inflammation markers of IL6 (>1.56pg/mL) was nearly doubled in HIV-infected individuals on (aPOR 1.84; 95%CI: 1.05-3.21) and not on (aPOR 1.94; 95%CI: 1.11-3.41) ART. Compared to HIV-uninfected, HIV-infected individuals on ART had >twice partial proportional odds (apPOR=2.30;p=0.004) of having non-clinically significant raised hsCRP levels(>1ug/mL); ART-naïve HIV-infected individuals had >double apPOR of having hsCRP levels indicative of increased heart disease risk(>3.9ug/mL;p=0.008).

**Conclusions:**

Although HIV status was associated with increased inflammatory markers, our results highlight reduced morbidity in those receiving ART and underscore the need of pro-actively extending these services to HIV-uninfected older adults, beyond mere provision at fixed clinics. Providing health services through regular community chronic disease screening would ensure health care reaches all older adults in need.

## Introduction

Older people (50+years) are at risk of chronic morbidity such as heart diseases, arthritis, diabetes and hypertension, associated with physiological changes with age [1-4], but these conditions remain often undiagnosed particular in resource poor settings. The disease burden may be exacerbated by both HIV and antiretroviral therapy (ART) [3,5-8], suggesting worse health outcomes in HIV-infected, especially those on ART, than in HIV-uninfected adults. Generally, the earlier a disease is diagnosed, the more likely it is that it can be cured or successfully managed. However, the association between HIV status and chronic morbidity, and possible benefit of regular access to general medical services within HIV treatment and care, remains little explored. Evidence on differential morbidity by HIV status from two studies was conflicting [9,10] and neither study include specific age-related morbidities in their outcome measure. 

Certain biomarkers are useful tools for predicting clinical events [11] and are increasingly employed in monitoring health, identifying individuals at risk and evaluating therapeutic interventions [12,13]. Cytokines are released in response to trauma, infection or inflammation and sustained elevation has been linked to age-associated conditions and increased mortality [13-16]. Cytokines of interest in chronic conditions of older age include Interleukin- 1 and 6 (IL1 and IL6), Tumor Necrosis Factor alpha (TNFα), and C-Reactive Protein (CRP)[14-17]. Little is known on the association of HIV and ART status with cytokine levels and age-related chronic morbidity.

Obesity is linked to chronic health problems such as cardiovascular diseases, diabetes and arthritis [12,17,18] and, similar to ageing, is characterised by chronic low-grade inflammation [17,19]. Cytokine levels, as inflammation, trauma or infection markers, are thus normally higher in obese [17,19] and HIV-infected individuals with advanced disease [20,21]. It is critical to understand the associations of obesity with cytokine levels increasingly used to measure health risks and explain individual health status, in both HIV-infected and HIV–uninfected older adults. 

We use data from a cross-sectional cohort of older people in a high HIV prevalence area to examine levels of chronic morbidity in HIV-infected and uninfected older adults and subsequent association of HIV and ART status with selected cytokines and BMI.

## Methods

### Ethics Statement

For the WOPS study, approval was first obtained from the local community through the Centre’s Community Advisory Board and then from the Biomedical Research Ethics Committee of the University of KwaZulu-Natal (Ref No. BF136/09). AC surveillance was approved in 2000 by this same committee, with annual re-certification (Ref Nos. E009/00 and BF233/09). Individual written informed consent was obtained from all WOPS and AC HIV surveillance participants. 

### Setting and data collection

Since 2000, demographic and health data have been collected by the Africa Centre (AC) on approximately 11 000 households in a geographically defined South African area. On 1 January 2010, there were 61 431 resident household members of whom about 7,900 (13%) were aged 50 years or above [9,22-24]. Within the household surveillance is a nested annual HIV surveillance, in which dried blood spot specimens are collected from eligible adults, for anonymized HIV testing [22-24], (www.africacentre.ac.za). 

The SAGE Well-being of Older People Study (WOPS) employed survey instruments adapted from the World Health Organization (WHO) Study on global AGEing and adult health (SAGE) [25,26] and was carried out within the AC surveillance area on a multi-stage random sample of individuals aged 50+years between March-August 2010 [9]. The main aim of SAGE-WOPS was to investigate the direct and indirect effects of HIV on the health of older adults [9]. For sample selection, all older adults resident within the AC surveillance area and falling into 3 categories namely: HIV-infected on ART, HIV-infected ART-naïve, and HIV-affected through co-residing with an HIV-infected individual, were identified through existing AC population databases. From all eligible individuals, random samples of 150 participants each for the first two groups and 300 from the third group were generated; participants were contacted through a home visit and enrolled into the study if they were willing and provided informed consent. Enrolment in each group was done until the required numbers (100 for each of the first two groups, 200 in the third group) were reached, giving a total of 400 participants. For purposes of this study, participants from the three groups comprising of HIV infected and HIV-uninfected individuals were grouped into mutually exclusive groups by their HIV/ART status. All contacted individuals agreed to participate in the study, and 22 individuals consented to the questionnaire and anthropometric measures only and not to blood collection, giving a sample size of 422 individuals in total. Participant HIV status was not disclosed during the WOPS interview. Geographical typology of the randomly selected individuals showed a distribution similar to the general distribution of the older adult population within the surveillance area, suggesting the representativeness of the sample. 

Demographic and health information was collected through face-to-face interviews. Participants were asked if they had been ever diagnosed with a named chronic morbidity, timing of diagnosis (last 6 months; >6-12months; >12months) and whether or not, for that named condition, they had received treatment in the last 2 weeks and/or 12 months. In addition, weight and height were measured by trained nurses, who also collected blood specimens for laboratory measured biomarkers of lipogram profile and cytokine levels (IL1, IL6, high sensitivity CRP (hsCRP) and TNFα). 

Information regarding HIV status was determined using the AC population based HIV surveillance and the Hlabisa HIV Treatment and Care programme databases (HHTCP) [27]; data from these two sources can be linked through use of the unique individual South African national identity number, name and sex [9,28,29]. HIV status information was subsequently updated after completion of the SAGE-WOPS interviews, as appropriate. From the HHTCP, we identified HIV-infected people and duration of therapy for those on ART. For those unknown to HHTCP, HIV status from the HIV surveillance prior- and post-WOPS were used to infer HIV status of participants at time of the WOPS study using the algorithm below:

• HIV-uninfected before and after WOPS = HIV-uninfected;
• HIV-infected before and after WOPS = HIV-infected;
• HIV-uninfected within a year prior to WOPS and unknown after WOPS = HIV-uninfected (*with an incidence rate of 0.5; (95% CI: 0.3-1.0) per 100 person years in adults 50+ years [30*] *we* *would* *expect* *at* *most* *only 1* *individual* *of* *the 51* *participants* *to* *have* *seroconverted* *within* *the* *year*); and
• HIV unknown before and after WOPS = unknown

### Variables

#### Self-reported current chronic morbidity

Based on responses to questions, “Have you been taking treatment for …… in the last 2 weeks?", including heart disease (angina), arthritis, stroke, hypertension, chronic lung disease, asthma and diabetes. This question was only asked from all participants reporting ever been diagnosed by a health care professional with any of the aforementioned conditions. 

#### BMI (indicator of obesity)

Categorized as per WHO recommendations: underweight: <18.5; normal: 18.5-<25; overweight (pre-obese): 25-<30; obese: 30-<40; morbidly obese: 40+ [31].

#### Cytokines

Although within the continuum of circulating cytokines, higher levels of cytokines are associated with chronic inflammation and morbidity, there is no defined cut-off point beyond which morbidity starts to increase. Consequently previous studies have chosen arbitrary cut-off points, employing a range of cut-offs from dividing the continuous distribution into tertiles and quartiles [13,15,20] , log-transformed continuous levels [13], median cut-off [15] or the lower cytokine detection limit [32]. For CRP, values >3µg/ml have been used to indicate increased risk of heart disease whilst values >8.5 indicate clinically relevant inflammation [32]. For this study we adopted cut-off points within range of existing studies, for uniformity and comparability of results. Categories were as follows;

IL1 (≤1.6, >1.6pg/mL); IL6 (≤1.56, >1.56-≤2.9, >2.9-≤5 and >5pg/mL); CRP (≤1, >1-3.9, >3.9-8.5, >8.5µg/mL) and TNFα (≤7.8, >7.8pg/mL).

#### Total cholesterol:high density lipoprotein (HDL) ratio (ratio of bad to good cholesterol)

Higher ratios are associated with increased cardiovascular disease risk [14,33].

• Males: ratio1(<3.4), ratio2(3.4-<5), ratio3(5-<9.6), ratio4(≥9.6)
• Females: ratio1(<3.3), ratio2(3.3-<4.4), ratio3(4.4-<7.1), ratio4(≥7.1)

### Laboratory procedures

All laboratory tests were conducted by a South African National Accreditation System (SANAS) certified laboratory (Global Clinical and Viral Laboratory). Tests were conducted using kits by BioVendor Research and Diagnostic Products, Czech Republic. Lower detection concentrations were 0.02ug/mL for hsCRP, 1.1pg/mL for IL1, 0.81pg/mL for IL6 and 5.0pg/mL for TNFα. Blood serum was used for determination of hsCRP, IL1 and TNFα levels and plasma for IL6.

### Analytical methods

Baseline characteristics were described using medians and IQRs (equality of medians tested for using Kwallis2 test [34]) for continuous variables and proportions with 95% CI for categorical variables. To assess the association of HIV and obesity with morbidity, ordinary logistic regression was employed. Because IL6 was categorized into an ordinal variable, ordered logistic regression [35,36] assessed the association between IL6 , HIV and obesity. Ordered logistic regression takes into account the hierarchy in the dependent variable categories assuming proportional odds (POR) and results in a single equation estimating the relationship between predictor variables and all levels of the dependent variable. Due to violation of proportional odds assumption, we examined associations of HIV and obesity with CRP using generalized ordered logistic regression which estimates multiple equations over the different CRP levels without assuming proportional odds, producing partial proportional odds ratios (pPOR) [36,37]. For IL1 (binary outcome) simple logistic regression was used. STATA 11.2 was used for all analyses (StataCorp LP).

## Results

Of the 422 older WOPS participants, 161 (38%) were HIV-uninfected, 108 (26%) were HIV-infected with at least a year on ART, 109 (26%) were HIV-infected ART-naïve and 44 (10%) had unknown HIV status with characteristics similar to those HIV-uninfected. Men comprised 25% of the 422 individuals (n=106). Median age for HIV-uninfected individuals was 10 years higher than for HIV-infected hence all analyses were age adjusted. As would be expected in this population and setting, few individuals reported currently or ever smoking or drinking (Table 1). 

**Table 1:** Baseline demographic and clinical characteristics of 422 older adults stratified by HIV status.

Characteristic		HIV-uninfected (161)			HIV-infected on ART (108)			HIV-infected ART naive (109)			^a^Total (422)		p-value
		N /median	%	(95% CI)/ IQR	N /median	%	(95% CI)/ IQR	N /median	%	(95% CI)/ IQR	N /median	%/ IQR	
Sex	Male	30	18.6	(12.6-24.7)	36	33.3	(24.4-42.3)	30	27.5	(19.1-36.0)	106	25.1	0.05
Age at interview		68		61-75	57		53-62	53		51-60	60	53-69	<0.001
Marital status	Married	40	24.8	(18.1-31.6)	36	33.3	(24.4-42.3)	33	30.6	(21.8-39.3)	120	28.5	
	Never been married	32	19.9	(13. 7-26.1)	33	30.6	(21.8-39.3)	43	39.8	(30.5-49.1)	116	27.6	<0.001
	Divorced/widowed	89	55.3	(47. -63.0)	39	36.1	(27.0-45.2)	32	29.6	(21.0-38.3)	185	43.9	
Employment	No	158	98.8	(97.0-1)	99	92.5	(87.5-97.5)	96	88.9	(82.9-94.9)	395	94.3	0.01
	Yes	2	1.3	(0-3.0)	8	7.5	(2.5-12.5)	12	11.1	(5.1-17.1)	24	5.7	
Main source of income	Grants	145	91.2	(86.8-95.6)	81	75.0	(66.8-83.2)	69	63.9	(54.8-73.0)	332	79.4	
	No source of income	7	4.4	(1.2-7.6)	9	8.3	(3.1-13.6)	18	16.7	(9.6-23.8)	36	8.6	<0.001
	Other	7	4.4	(1.2-7.6)	18	16.7	(9.6-23.8)	21	19.4	(11.9-27.0)	50	12.0	
BMI categories	Underweight	4	2.6	(0.1-5.1)	7	6.6	(1.8-11.4)	12	11.2	(5.2-17.2)	25	6.1	
	Normal	46	29.9	(22.6-37.1)	40	37.7	(28.4-47.0)	30	28.0	(19.5-36.6)	127	31.0	
	Overweight	45	29.2	(22.0-36.5)	37	34.9	(25.8-44.1)	35	32.7	(23.8-41. 7)	127	31.0	0.04
	Obese	48	31.2	(23.8-38.5)	17	16.0	(9.0-23.1)	24	22.4	(14.5-30.4)	105	25.6	
	Morbidly obese	11	7.1	(3.0-11.2)	5	4.7	(0.7-8. 8)	6	5.6	(1.2-10.0)	26	6.3	
Smoking	Never smoked	117	73.1	(66.2-80.0)	99	91.7	(86.4-96.9)	74	67.9	(59.1-76.7)	324	77.0	
	Past smoker	24	15.0	(9.4-20.6)	6	5.6	(1.2-9.9)	16	14.7	(8.0-21.4)	49	11.6	0.001
	Current smoker	19	11.9	(6.83-16.9)	3	2.8	(0-5.9)	19	17.4	(10.3-24.6)	48	11.4	
Alcohol	Never drank	94	58.75	(51.1-66.4)	82	75.9	(67.8-84.1)	64	58.7	(49.4-68.0)	269	63.9	
	Past drinker	41	25.63	(18.8-32.4)	14	13.0	(6.6-19.4)	33	30.3	(21.6-39.0)	94	22.3	0.01
	Current drinker	25	15.6	(9.96-21.3)	12	11.1	(5.1-17.1)	12	11.0	(5.1-16.9)	58	13.8	
Composite health score	continuous	46.7		43.1-53.1	52.3		47.9-57.4	48.6		44.1-54.1	49.22	45-55	0.001
	Healthy	47	29.2	(22.1-36.3)	59	54.6	(45.2-64.1)	41	37.6	(28.5-46.8)	159	37.7	<0.001
	Unhealthy	114	70.8	(63.7-77.9)	49	45.4	(35.9-54.8)	68	62.4	(53.2-71.6)	263	62.3	
Ever diagnosed with morbidity	No	38	23.6	(17.0-30.2)	39	36.1	(27.0-45.2)	29	26.6	(18.3-5.0)	121	28.7	0.12
	Yes	123	76.4	(69.8-83.00)	69	63.9	(54.8-73.0)	80	73.4	(65.0-81.7)	301	71.3	
Current morbidity	No	70	43.5	(35.8-51.2)	66	61.1	(51.9-70.4)	54	49.5	(40.1-59.0)	215	51.0	0.03
	Yes	91	56.5	(48.8-64.2)	42	38.9	(29.6-48.2)	55	50.5	(41.0-59.9)	207	49.1	
Morbidity in the last 12 months	No	52	32.3	(25.0-39.6)	52	48.2	(38.7-57.6)	48	44.0	(34.6-53.4)	173	41.0	0.04
	Yes	109	67.7	(60.4-75.0)	56	51.9	(42.4-61.4)	61	56.0	(46.6-65.4)	249	59.0	

^a^ total includes 44 participants with unknown HIV status (described in text)

### Self-reported morbidity

**Figure 1:**
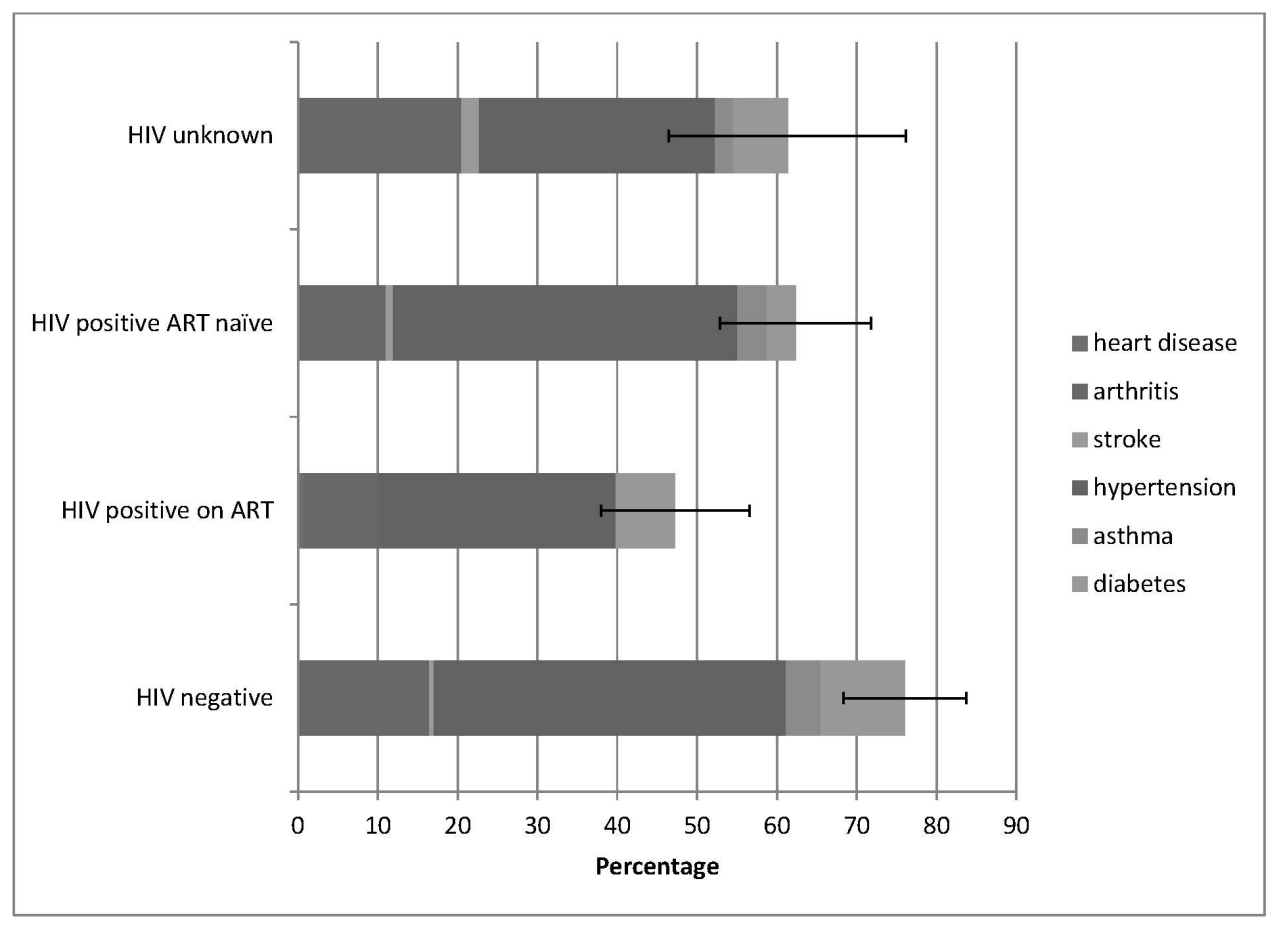
Proportion with 95% confidence intervals of disease specific chronic morbidity in 422 older adults stratified by HIV status.

Of the 422 participants, 124 (29.4%; 95% CI: 25.0-33.8) reported never having been diagnosed with any chronic condition (Table 1) whilst 169(40.1%; 95% CI: 35.4-44.7) and 100(23.7%; 95% CI: 19.6-27.8) reported diagnosis with one and two conditions, respectively; 29(6.9%) individuals had more than two conditions. Significantly more HIV-uninfected and HIV-infected ART-naïve participants than HIV-infected participants receiving ART reported current morbidity i.e. receiving therapy for either one of heart disease, arthritis, stroke, hypertension, asthma or diabetes (Figure 1) (p=0.033). Specific current morbidities are illustrated using Figure 1. 

### Anthropometry

Median BMI was highest in those HIV-uninfected compared to HIV-infected (28.1 vs 25.3 (p=0.057)); obesity was more frequent among HIV-uninfected than among HIV-infected on ART and ART-naïve (Table 1).

### Cytokines

Overall, there was little variation in median IL6 by HIV status (Table 2). For TNFα, only 7(1.8%) participants had elevated levels, with medians similar across all HIV status strata (p=0.231) therefore TNFα was not assessed further. Significantly more HIV-uninfected people had IL1 levels above 1.6µg/mL than those HIV-infected ART-naive (p=0.003), although the medians were the same across groups (Table 2). There was a trend towards highest CRP levels (>8.5pg/ml) in those HIV-infected, with a statistically significant difference in HIV-infected ART-naive compared to HIV-uninfected. Obese/morbidly-obese participants had increased CRP levels in both the median and categorized analyses (Table 2).

**Table 2:** Cytokine (IL1, IL6, CRP and TNFα) levels of 422 old adults stratified by HIV status and BMI levels.

HIV status		HIV negative		HIV positive on ART		HIV positive ART naive		Total
		Median/N	% (95% CI)/(IQR)	Median/N	% (95% CI)/(IQR)	Median/N	% (95% CI)/(IQR)	Median/N (IQR/95%CI)
IL1		1.6	(1.6-1.6)	1.6	(1.6-1.6)	1.6	(1.6-1.6)	1.6 (1.6-1.6)
	<=1.6	123	83.1 (77.0-89.2)	100	92.6 (87.6-97.6)	94	96.9 (93.4-1.0)	353 (90.1)
	>1.6	25	16.89 (10.8-23.0)	8	7.4 (2.4-12.4)	3	3.1 (0-6.6)	39 (10.0)
IL6		1.94	(1.6-2.6)	2.5	(2.0-3.1)	2.6	(2.0-3.2)	2.4 (2.1-2.6)
	<=1.56	70	47.3 (39.2-55.4)	38	35.2 (26.1-44.3)	36	37.1 (27.4-46.8)	157 (40.1)
	>1.56-2.9	20	13.5 (8.0-19.1)	24	22.2 (14.3-30.1)	19	19.6 (11.6-27.6)	68 (17.4)
	>2.9-5	26	17.6 (11.4-23.7)	21	19.4 (11.9-27.0)	19	19.6 (11.6-27.6)	73 (18.6)
	>5	32	21.6 (15.0-28.3)	25	23.2 (15.1-31.2)	23	23.7 (15.2-32.3)	94 (24.0)
CRP		3.7	(2.5-4.1)	4.2	(3.5-5.8)	4.3	(2.6-6.5)	3.9 (3.2-4.3)
	<=1	31	21.2 (14.6-27.9)	16	15.1 (8.2-22.0)	21	21.7 (13.4-29.9)	78 (20.1)
	>1-3.9	52	35.6 (27.8-43.4)	33	31.1 (22.3-40.0)	25	25.8 (17.0-34.6)	122 (31.4)
	>3.9-8.5	39	26.7 (19.5-33.9)	24	22.6 (14.6-30.7)	19	19.6 (11.6-27.6)	92 (23.7)
	>8.5	24	16.4 (10.4-22.5)	33	31.1 (22.3-40.0)	32	33.0 (23.6-42.4)	96 (24.7)
BMI		Normal		Overweight		Obese/ morbidly obese		Total
		Median/N	% (95% CI)/(IQR)	Median /N	% (95% CI)/(IQR)	Median /N	% (95% CI)/(IQR)	Median/N (IQR/95%CI)
IL1		1.6	(1.6-1.6)	1.6	(1.6-1.6)	1.6	(1.6-1.6)	1.6 (1.6-1.6)
	<=1.6	134	90.5 (85.8-95.3)	107	93.0 (88.4-97.7)	103	87.3 (81.2-93.3)	353 (90.1)
	>1.6	14	9.5 (4.7-14.2)	8	7.0 (2.3-11.6)	15	12.7 (6.7-18.8)	39 (10.0)
IL6		2.5	(2.0-3.2)	2.5	(1.7-3.2)	2.08	(1.6-2.6)	2.4 (2.1-2.6)
	<=1.56	58	39.2 (31.3-47.1)	47	40.9 (31.8-49.9)	51	43.2 (34.2-52.2)	157 (40.1)
	>1.56-2.9	24	16.2 (10.2-22.2)	17	14.8 (8.3-21.3)	25	21.2 (13.8-28.6)	68 (17.4)
	>2.9-5	26	17.6 (11.4-23.7)	28	24.4 (16.4-32.3)	17	14.4 (8.0-20.8)	73 (18.6)
	>5	40	27.0 (19.8-34.2)	23	20.0 (12.6-27.4)	25	21.1 (13.8-28.6)	94 (24.0)
hsCRP		2.5	(1.8-4.0)	3.2	(2.5-3.9 )	6.15	(4.8-6.9)	3.9 (3.2-4.3)
	<=1	46	31.3 (23.8-38.8)	17	14.9 (8.3-21.5)	13	11.2 (5.4-17.0)	78 (20.1)
	>1-3.9	39	26.5 (19.4-33.7)	54	47.4 (38.1-56.6)	27	23.3 (15.5-31.0)	122 (31.4)
	>3.9-8.5	27	18.4 (12.1-24.67)	20	17.5 (10.5-24.6)	43	37.1 (28.2-45.9)	92 (23.7)
	>8.5	25	23.8 (16.9-30.7)	23	20.2 (12.8-27.6)	33	28.5 (20.2-36.7)	96 (24.7)

Abbreviations: BMI: body mass index (measured as weight in kilograms divided by the square of height in meters), IL1: Interleukin 1, IL6: Interleukin 6, hsCRP: high sensitivity C-reactive protein, ART: antiretroviral therapy

### HIV status, obesity and morbidity

**Figure 2:**
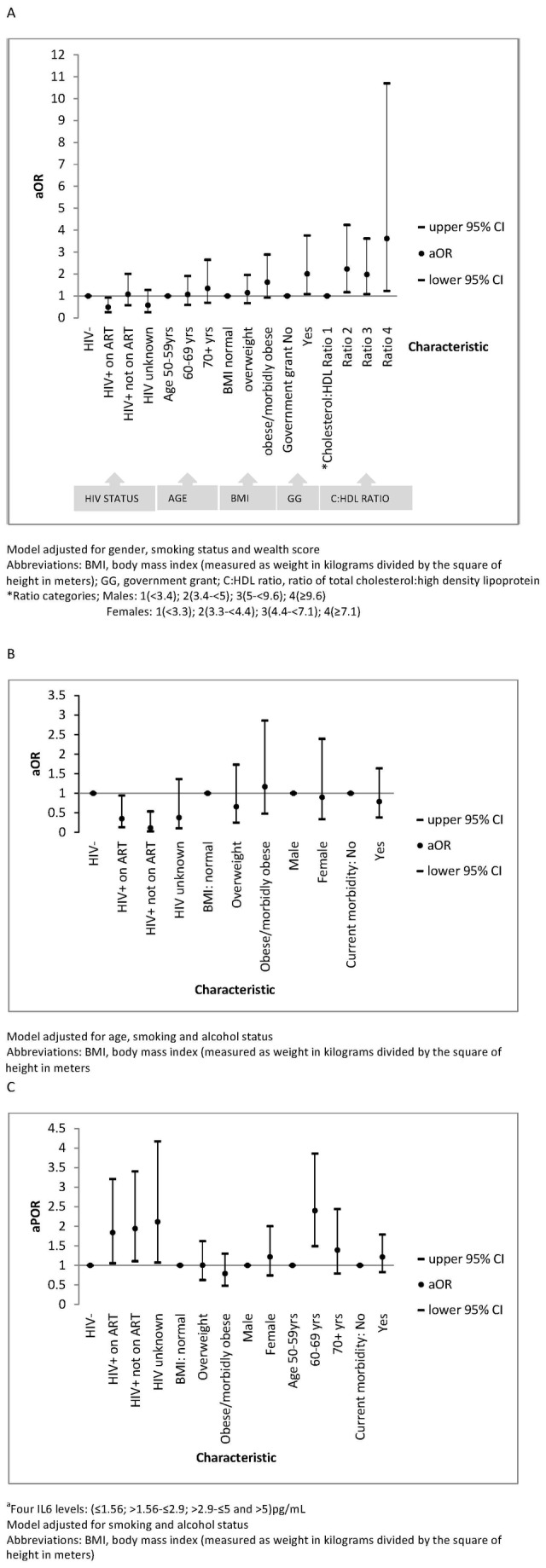
a. Logistic regression model of factors associated with current chronic morbidity in 422 older adults. b. Logistic regression of factors associated with IL1 levels in 422 older adults. c. Ordered logistic regression model for factors associated with #IL6 levels in 422 older adults.

Controlling for factors known to be associated with ill health (age, sex, smoking and wealth quintile), HIV-infected older adults on ART were significantly less likely (OR=0.49, p=0.027) to report current morbidity than HIV-uninfected adults (Figure 2a). Cytokine levels were not significantly associated with morbidity. In a model including obesity marker (BMI) but not ratio of good:bad (HDL) cholesterol, there was borderline association between being obese/morbidly-obese and current morbidity (aOR=1.75; 95%CI: 1.0-3.0). However, including cholesterol:HDL ratio in the model, BMI lost its significance whilst higher levels of this ratio significantly increased the odds of current morbidity(Figure 2a). Cholesterol:HDL ratio was associated with BMI, with normal BMI category having only 4.0% and those obese 11.7% with ratio4. Of the obese/morbidly-obese, only 10.8% had ratio 1 compared to 28.7% of those with normal BMI. 

### HIV status, obesity and cytokine levels

#### IL1

Adjusting for lifestyle factors (smoking and alcohol), age and gender, compared to HIV-uninfected, the odds of having IL1 levels >1.6pg/ml was lower by 65% (aOR=0.35; 95%CI: 0.13-0.94) and 89% (aOR=0.11; 95%CI: 0.24-0.54) for HIV-infected on ART and HIV-infected ART-naïve, respectively (Figure 2b). 

#### IL6

In adjusted ordered logistic regression, compared to HIV-uninfected, the proportional odds (aPOR) of having low IL6 levels was nearly twice as high in HIV-infected individuals both on ART and ART naïve. The proportional odds of having elevated IL6 levels (aPOR 2.40; 95%CI: 1.49-3.86) was higher in those aged 60-69 years than in those aged 50-59 years. A non-significant increased odds was observed in individuals aged 70+years (aPOR=1.39; p=0.248) (Figure 2c). 

Cholesterol:HDL ratio and BMI were not significantly associated with IL1 and IL6 cytokine levels (IL1 p=0.675, IL6 p=0.681).

#### CRP

Compared to HIV-uninfected, HIV-infected individuals on ART had more than twice the partial proportional odds (apPOR=2.30; p=0.004) of having slightly raised hsCRP levels(>1ug/mL-levels that have been associated with non-clinically significant inflammation) whilst ART-naïve HIV-infected individuals had more than double apPOR of having hsCRP levels indicative of increased cardiovascular disease risk (>3.9 ug/mL) (p=0.008). HIV infection and cholesterol:HDL ratio 4 were the only independent factors associated with very high levels of hsCRP (>8.5ug/mL – levels that may signify clinically relevant inflammation); the likelihood was even higher in ART-naïve HIV-infected participants(Table 3). 

**Table 3:** Generalised ordered logistic regression model for factors associated with elevated CRP levels in old adults n=422.

CRP levels	Odds Ratio	P-value	95% Confidence interval	
**<=1pg/mL**				
HIV-				
HIV+ on ART	2.30	0.004	1.31	4.06
HIV+ ART naive	1.03	0.93	0.51	2.08
HIV unknown	0.83	0.61	0.42	1.66
BMI normal				
Overweight	2.54	0.005	1.33	4.85
Obese/morbidly obese	3.72	<0.001	1.81	7.63
Age 50-59years				
60-69 years	1.06	0.87	0.54	2.05
70+ years	1.27	0.41	0.73	2.21
**>1-3_9pg/mL**				
HIV-				
HIV+ on ART	2.30	0.004	1.31	4.06
HIV+ ART naive	2.30	0.008	1.25	4.24
HIV unknown	0.83	0.61	0.42	1.66
BMI normal				
Overweight	0.78	0.37	0.46	1.33
Obese/morbidly obese	2.78	<0.001	1.58	4.89
Age 50-59years				
60-69 years	2.09	0.009	1.21	3.61
70+ years	1.27	0.41	0.73	2.21
**>3_9-8_5pg/mL**				
HIV-				
HIV+ on ART	2.30	0.004	1.31	4.06
HIV+ ART naive	2.81	0.002	1.47	5.38
HIV unknown	0.83	0.61	0.42	1.66
BMI normal				
Overweight	0.80	0.48	0.43	1.48
Obese/morbidly obese	1.41	0.27	0.77	2.61
Age 50-59years				
60-69 years	1.43	0.23	0.80	2.57
70+ years	1.27	0.41	0.73	2.21
**Across all levels of hsCRP**				
^a^Cholesterol:HDL ratio 1				
Ratio 2	1.12	0.70	0.64	1.96
Ratio 3	1.47	0.16	0.86	2.53
Ratio 4	2.51	0.04	1.03	6.09

Adjusted for gender, current morbidity, smoking and alcohol status

^a^ Ratio categories; Males: 1(<3.4), 2(3.4-<5), 3(5-<9.6), 4(≥9.6)

Females: 1(<3.3), 2(3.3-<4.4), 3(4.4-<7.1), 4(≥7.1)

Abbreviations: BMI: body mass index (measured as weight in kilograms divided by the square of height in meters), IL1: Interleukin 1, IL6: Interleukin 6, hsCRP: high sensitivity C-reactive protein, ART: antiretroviral therapy

Although all BMI levels above normal increased the odds of having hsCRP levels>1ug/mL, being obese/morbidly-obese nearly tripled the likelihood of having hsCRP levels associated with increased cardiovascular disease risk (>3.9ug/mL) beyond which BMI was not associated with higher CRP levels (Table 3). Compared to those aged below 60 years, those aged 60-69years were twice as likely to have elevated hsCRP levels. Having cholesterol:HDL ratio4 was associated with three times more proportional odds of having elevated hsCRP levels across all CRP levels (Table 3). 

Current morbidity was not associated with cytokines levels.

## Discussion

Older HIV-infected adults face both chronic conditions of ageing and HIV [1,4,38,39], with data suggesting that HIV treatment may exacerbate chronic conditions associated with aging [2,3,6,40]. There is lack of reliable data in Africa regarding associations of HIV, obesity and age-related morbidity especially comparing morbidity by HIV and ART status. This study contributes to knowledge by being the first to demonstrate, in a rural African setting, the possibility of less current chronic morbidity in HIV-infected older adults receiving ART compared to HIV negatives. Could this be due to access to ART and/or health services?

We previously reported a higher WHO composite health score [a health measure collating an individual’s levels of difficulty in eight health domains (mobility, self-care, affect, vision, pain/discomfort, sleep/energy, interpersonal activities, and cognition)] amongst HIV-infected than in HIV-uninfected individuals, not accounting for ART status [9]. We now confirm this previous finding with more in-depth health measures and highlight differences by ART status. The fact that we previously report a higher composite health score using the same study population reduces the possibility that chronic morbidity in HIV-infected individuals remains undiagnosed or is misdiagnosed as HIV-related morbidity. It is likely that morbidity in HIV-infected older adults receiving ART is reduced through regular screening and treatment during frequent routine HIV clinic visits. In this setting, in accordance with the South African National HIV treatment guidelines, HIV positive individuals on ART visit the HIV clinics each month to consult with the HIV nurse and collect ART pills. Additionally patients receive adherence and health counseling during these monthly visits. Our findings here could suggest benefit of enhanced access to health care in HIV positive older adults through sustained frequent utilization of health care services for HIV treatment and care. In HIV positive individuals pre-ART, we did not observe morbidity differences from those HIV negative possibly due to the relatively infrequent clinic visits pre-ART since HIV positive individuals in routine HIV care and not yet eligible for ART are advised to visit the clinic once in six months.

Although elevated cytokine levels have been associated with increased cardiovascular and diabetes morbidity [17,19] it remains unknown whether the elevated cytokine levels result in morbidity or immune inflammatory response due to morbidity results in elevated cytokines. Our finding of lower morbidity in HIV-infected adults receiving ART and of the increased odds of elevated hsCRP and IL6 levels in this group may suggest that these cytokines may be associated more with chronic HIV rather than with other existing chronic morbidity. Compared to those HIV-uninfected, HIV-infected individuals on ART had nearly twice the odds of having elevated IL6 levels and more than twice the odds of elevated hsCRP levels, indicating immune inflammatory response. Similar elevated cytokine levels in HIV-infected adults have been reported from studies in resource-rich countries focused on HIV-infected people only, however these did not make comparisons with HIV-uninfected adults nor explored the association with ageing-morbidity [20,21]. Our study is the first to assess how in an African black population, controlling for age differences across HIV strata, cytokine levels differ by HIV and ART status and how these levels associate with chronic morbidity during ART. Although previous studies, not accounting for HIV status, report higher morbidity in individuals with elevated cytokine levels, they acknowledge that clinical mechanisms and significance of this phenomenon remains unclear [13-16].

Our results of lower morbidity in HIV-infected on ART, but not HIV-infected ART-naïve, than in HIV-uninfected older adults irrespective of high HIV-associated cytokine levels, may highlight the likelihood that even in the absence of co-morbid conditions, cytokine levels in HIV-infected adults do not return to pre-HIV infection levels despite ART and cytokine levels may not be ideal markers for chronic morbidity in such populations. Some studies have suggested that soluble cytokine receptors released in response to elevated cytokine levels, are more stable in circulation over time and hence might be more reliable markers of chronic inflammation than cytokines [13]. Longitudinal studies are needed to elucidate associations between HIV status and cytokine levels and how these relate to incident chronic morbidity and to explain mechanisms leading to morbidity decline in HIV-infected older adults on ART and to explain mortality differentials from chronic morbidity by HIV status. Within lifelong exposure to ART, vigilant monitoring of liver and kidney toxicities is required as these would negate the realized benefits.

Our results show that in this population with high obesity levels, it is the ratio of bad:good fat (cholesterol) ratio, a marker of cardiovascular disease risk, that is associated with high morbidity rather than BMI per se. In an analysis adjusted for this ratio, BMI ceased being an independent factor of morbidity, with the odds of morbidity nearly quadrupling in individuals within the highest ratio quartile possibly suggesting that total cholesterol:HDL ratio may be a stronger indicator of morbidity than BMI. Although BMI was not associated with morbidity when accounting for the ratio of bad:good fat, being obese/morbidly obese was associated with high hsCRP levels suggestive of increased inflammation and cardiovascular disease risk. Studies from developed countries have also shown increased inflammatory response in obese people [17,19] but literature from African populations is scarce.

Our results underscore the need of extending health care services to HIV-uninfected older adults, which need to go beyond mere provision at fixed clinics. Bringing health services to older adults through regular community chronic disease screening would ensure health care reaches all older adults in need, and could translate to considerable health benefits. 

Our cross-sectional study has limitations, and we cannot assume causality in our associations but highlight possible associations which could be further elucidated in longitudinal cohort studies. Due to the complexities surrounding data collection of self-reported non-HIV medications for non-communicable chronic diseases, we did not collect information on the type of drugs that participants were taking for other age-related chronic conditions. Hence we could not assess the association of these medications with cytokine levels and morbidity by HIV and ART status. Although we cannot rule out the role of survivor bias, if the observed reduced reported morbidity in HIV-infected receiving ART was purely due to survivor bias we would also expect even larger morbidity decreases amongst the HIV-infected ART naïve group, which is not the case. Although our data was self-reported, we assumed that any unreliability of self-reports occurred equally across groups resulting in non-differential bias which does not affect validity of our results. This assumption was based on the fact that there is no evidence supporting the likelihood of over-reporting current morbidity amongst HIV-uninfected, but not infected, individuals. It is likely that HIV-infected individuals may over-report morbidity due to their knowledge of the underlying HIV infection. Furthermore both HIV-infected and HIV-uninfected participants were identified from the community via the longitudinal demographic surveillance system rather than from health care facilities, and thus would have reduced selection bias. Although our sample size is small, limiting the extent to which we could detect differences between groups, the fact that despite this we were able to detect significant differences between HIV-infected participants receiving ART and those HIV-uninfected possibly points towards an even larger morbidity difference had we used a larger sample size with tighter confidence intervals. As such the sample size issue does not nullify our results but rather confirms the strength of existing associations between morbidity prevalence and HIV-infection and ART. 
